# Toward Improved Triadic Functioning: Exploring the Interactions and Adaptations of Coaches, Parents and Athletes in Professional Academy Soccer Through the Adversity of COVID-19

**DOI:** 10.3389/fpsyg.2021.609631

**Published:** 2021-05-19

**Authors:** James Maurice, Tracey J. Devonport, Camilla J. Knight

**Affiliations:** ^1^Sport and Physical Activity Research Centre, University of Wolverhampton, Walsall, United Kingdom; ^2^School of Sport and Exercise Sciences, Swansea University, Swansea, United Kingdom

**Keywords:** academy, coaches, parents, players, collaboration, soccer

## Abstract

On March 23rd, 2020, elite soccer academies in the UK closed in compliance with the government enforced lockdown intended to contain the COVID-19 pandemic. This forced parents, players, and coaches to reconsider how they interacted with, and supported, one another. The aims of the present study were (a) to explore the perceptions of players, parents, and coaches (i.e., the athletic triangle) regarding how they interacted and collaborated with one another during the COVID-19 pandemic to support wellbeing and performance, and; (b) to identify opportunities to enhance workings of those within the athletic triangle resulting from adaptions made following enforced lockdown. Using an interpretive description methodology, semi-structured interviews were conducted with five coaches, six players, and six parents from an English elite academy soccer club. Interviews were analyzed using thematic analysis. Findings highlighted (a) the importance of support and the different means of communication used between members of the athletic triangle to facilitate such support; (b) the increased understanding of each member of the athletic triangle, leading to enhanced relationships, and; (c) how members of the athletic triangle adapted practice to facilitate relationship development during the pandemic and beyond. The identification of these considerations has implications for coach and parent education initiatives to allow for optimal functioning of the athletic triangle as elite academy soccer clubs return from lockdown. These include (a) the importance of continued communication between coach, athlete and parent; (b) increasing understanding of each individual within the athletic triangle; and (c) utilizing key interpersonal and technological skills learnt during the lockdown to further facilitate engagement within the athletic triangle.

## Introduction

On March 11th, 2020, the World Health Organization (WHO) declared the outbreak of COVID-19 a pandemic, meaning that the disease had spread worldwide. On the March 23rd, in response to the pandemic, the UK government introduced an initial three-week full-scale lockdown across the country. Guidelines stated that people would only be able to leave their home to shop for basic necessities, partake in one form of exercise a day, provide care for a vulnerable person, or to travel to and from essential work that could not be completed from home (Gov.UK, 2020).

On March 13th, the English Premier League and English Football League took the decision to suspend all domestic league and cup competitions, which also included the cessation of training for all clubs across the country (Premier League, 2020). Not only did this impact those at the first team level of soccer, both in the men and women’s game (Clarkson et al., 2020), but it also affected the thousands of young people involved in academy soccer. As lockdown continued, the COVID-19 pandemic left players and their families, as well as coaches and support staff, having to adapt to prolonged periods of social distancing. This also meant daily face-to-face interactions between players, coaches, and parents were prevented.

Within talent development pathways, such as soccer academies, it is evident that coherence between key stakeholders (e.g., coaches, athletes, parents, etc.) is a key factor influencing effectiveness (Martindale et al., 2007; Henriksen, 2015). As Martindale et al. (2005) argued, clarity and consistency of message are needed between the key stakeholders of young athletes, specifically coaches and parents, if the child’s potential is to be reached. Thus, strategies such as frequent communication and regular in-person interactions are often adopted within academies to share such messages. Unfortunately, the unprecedented situation presented by lockdown measures meant that those working within soccer academies suddenly and rapidly had to develop new ways of maintaining such clarity and consistency of message between coaches, parents, and players.

The nature and quality of interactions and relationships that develop between parents, coaches, and young people can have consequences not only in maintaining players’ technical development, but also facilitating optimal psychological, social, and performance outcomes (Davis and Jowett, 2010; Brustad, 2011). For example, research examining parent and coach initiated motivational climates within youth sport indicated that both motivational climates were significantly related to children’s psychological outcomes such as self-esteem, trait anxiety, and autonomous regulation, as well as sporting behavior (O’Rourke et al., 2014; Davies et al., 2016).

However, it is recognized that interactions between these individuals (i.e., the members of the ‘athletic triangle’; Smith et al., 1989) can be complex (Knight et al., 2017). For example, Clarke and Harwood (2014) identified that for parents within elite academy soccer, negotiating the distribution of power and responsibility with their son’s coach was a challenge, as was understanding what was expected of them. Such complexity is exacerbated as the requirements of parents and coaches, and consequently the interactions that occur between parents, coaches, and players, differ depending upon player’s age and stage of development (Wylleman and Lavallee, 2004). For instance, children may first enter soccer academies at the age of 8 or 9 years. At this age they are constantly playing, getting together and learning how to get along with their peers, however, they will still seek refuge with their parents if they feel insecure (Erikson, 1982). Parents of children within this age group take on a leadership role and are often highly involved in their child’s life, both within and beyond sport (Fraser-Thomas et al., 2008). Moreover, children of this age, place great value upon their parents’ guidance and support (Hoyle and Leff, 1997; Furusa et al., 2020) and thus during challenging times, such as the COVID-19 pandemic and associated lockdown, they are likely to be more heavily reliant upon their parents (Nickerson and Nagle, 2005).

In contrast, as children move into adolescence, they are looking to achieve more mature relationships with their peers, whilst also seeking independence from parents and older adults (Rice, 1998). At this stage, coaches begin to have more involvement over the child’s sporting development (Bloom, 1985), with parents becoming less involved in the provision of sporting advice at this age (Knight et al., 2010). However, parents remain critical in their continued provision of emotional support, helping their child to manage obstacles and challenges, which are likely to be apparent during the COVID-19 pandemic (Holt and Dunn, 2004; Holt and Mitchell, 2006; Knight et al., 2010). The impact of government mandated behavior change requirements during COVID-19 comes at an important time for those children in late childhood and young adolescence (herein collectively referred to as players) who are experiencing profound psychological and socioemotional transformation (Orben et al., 2020; Urbina-Garcia, 2020). As players experience such psychological and transformational challenges, it is likely that the dynamic interplay between members of the athletic triangle would remain critical during lockdown.

Individuals within the athlete triangle can influence each other in numerous ways. Specifically, Sprecher et al. (2002) argued that network members can affect the relationship quality of the dyadic relationship through three different processes: opportunity, information, and support. Opportunity refers to the experiences or possibilities that one individual provides the dyad to initially meet, build, and then sustain their relationship. Information refers to the many different pieces of information that an individual can provide to the dyad. Support refers to how an individual may assist in providing care or understanding to a member or both members of the dyadic relationship. Applying this theory to understand the influence of parents on coach-athlete relationships, Jowett and Timson-Katchis (2005) highlighted the importance of parents in providing key information regarding nutrition, wellbeing, and other activities, as well as providing opportunities through financial backing and providing transport. However, the researchers also argued that parents could be more influential if they were given a chance to share more information, provide further opportunities to their child and coach, and were made more aware of the difficult transitions experienced through the development pathway. In contrast to daily interactions at academies, where players and coaches communicate and players may then pass information on to their parents, during the pandemic, much of the interaction would have to occur between coaches and parents who would then distil this information to players. Therefore, it could be argued that during the pandemic, the importance of the quality of the relationship between parents and coaches was further enhanced as parent-coach interactions would not only play a key role in the maintenance and development of the coach-athlete relationship, but the continued holistic development of the player.

Existing literature examining parent-coach interactions within sport shows that positive interactions between coaches and parents are associated with superior experiences for young athletes (Wolfenden and Holt, 2005). For instance, there is evidence of higher enjoyment levels, reduced perceptions of pressure and anxiety, and smoother transitions (Wolfenden and Holt, 2005; Lauer et al., 2010; Knight and Holt, 2014). Findings have also shown that positive parent-coach interactions are beneficial in facilitating coaches’ work with young athletes (Gould et al., 2008), as well as enhancing parents’ involvement in their children’s sporting lives (Knight and Gould, 2017). For instance, in their study examining parental involvement in youth tennis, Knight and Holt (2014) identified that optimal involvement is underpinned by parents seeking to understand and enhance their children’s journey. Key to this was developing an understanding emotional climate, in which they demonstrated an understanding of their child and their child’s sport. One of the key strategies to develop such understanding was through maintaining a strong parent-coach relationship, enabling parents to obtain more guidance from coaches.

However, research examining parental stressors within a youth soccer academy has identified that the quality of communication from coaches can be a key stressor for parents in trying to navigate their way through academy soccer (Harwood et al., 2010). Specifically, parents have indicated that they can feel ‘excluded from the communication loop, unable to assist in their sons’ development’ (Harwood et al., 2010, p. 52). Furthermore, parents have highlighted that uncertainty regarding how to approach coaches can also be a cause for concern within academy soccer (Clarke and Harwood, 2014). Consequently, it appears that, while parent-coach communication is needed for optimal involvement from parents, such communication is not always forthcoming and, in fact, some coaches actively avoid contact with parents or limit their interactions with them (Knight and Gould, 2017).

Given the criticality of effective interactions between members of the athletic triangle, particularly communication, in enhancing not only players but also parents and coaches’ experiences and wellbeing, understanding the impact of enforced social distancing on such interactions is important. Particularly, identifying strategies that can be used to maintain interactions when in-person engagement is not possible will not only provide pertinent insights regarding how to maintain relationships during this pandemic, but also provide insights that may be beneficial to enhance relationships once social distancing requirements are removed. As such, the aims of the present study were (a) to explore the perceptions of players, parents, and coaches (i.e., the athletic triangle) regarding how they interacted and collaborated with one another during the COVID-19 pandemic to support wellbeing and performance, and; (b) to identify opportunities to enhance workings of those within the athletic triangle resulting from adaptions made following enforced lockdown.

## Materials and Methods

### Methodological Approach

An Interpretive Description (ID; Thorne, 2016) methodology was employed for this study to gain insight into the way members of the athletic triangle have collaborated with one another during COVID-19, and to identify learning opportunities to facilitate effective relationships following the pandemic. Developed by Thorne et al. (1997), the purpose of ID is to understand complex disciplinary questions and generate new knowledge for use in applied research settings (Thorne, 2016). ID utilizes an inductive analytical approach to identify themes and patterns within participants’ subjective perceptions and experiences but acknowledges that the researcher may possess prior knowledge in the area being explored which may influence data collection and analysis (Thorne, 2016). Therefore, ID was deemed appropriate for the current study for two reasons: (1) It sought to understand a complex question from the perspective of three different participant groups (i.e., parents, coaches, and players), and; (2) the study aimed to produce practical, applicable information intended to enhance collaborative working of those within the triad.

Consistent with a constructivist philosophy, ID recognizes the constructed and contextual nature of human experience whilst accepting that there can be shared realities across individuals (Thorne, 2016). With regards to its ontological orientation, ID is naturalistic, assuming that the development of knowledge results from the interaction between the researcher and the object being researched. Furthermore, due to the multiple realities that will be encountered, no *a priori* theory would be able to encompass such knowledge, and therefore, a theory must develop from or be grounded in the data. For this reason, this study does not claim to represent a definitive truth, but rather a ‘tentative truth claim’ (Thorne et al., 2004).

### Participants

Participants were recruited from a category one elite soccer academy in England^1^. Purposeful maximum variation sampling (Patton, 2002) was used to identify parents, players, and coaches across the academy who could provide insights into how they interacted and communicated during the COVID-19 lockdown. Specifically, the following criteria were applied: (a) a player within the under 9 to under 16 age groups; (b) a parent of a player within the under 9 to under 16 age groups and (c) full time coach within the under 9 to under 16 age groups. Overall, the sample consisted of 17 participants, of which there were five coaches, six parents, and six players. Coaches (all male) ranged in age from 26 to 42 years (*M* = 34.4) and had been coaching in academy soccer for an average of 7.4 years. Parents (three fathers and three mothers) ranged in age from 34 to 52 years (*M* = 43.8) and had been involved in academy soccer for an average of 6.3 years. Players ranged in age from 8 to 15 years (*M* = 13) and had been involved in academy soccer for an average of 5.8 years, (ranging from 2 to 8 years). Of note, the parents and players were each in dyads (i.e., each parent participant, was the parent of one of the player participants). Of the 17 participants, six were located within the foundation phase (FP: Under 9 (U9) to Under 12 (U12) and 11 within the youth development phase (YDP: Under 13 (U13) to Under 16 (U16)). To maintain anonymity, it was not possible to identify the specific age group of each participant. Rather participants were classified as early (E; first two years of the age group) or late (L; last two years of the age group) FP or YDP. Each participant was given a pseudonym, which is used throughout the manuscript. Further participant information is presented in Table 1.

**TABLE 1:** Participant information.

Participant Number	Name	Role	Age Group
1	Liam	Coach	YDP-L
2	David	Coach	YDP-L
3	Oliver	Coach	FP - E
4	John	Parent	YDP-E
5	Daniel	Player	YDP-E
6	Rachel	Parent	YDP-L
7	Harry	Player	YDP-L
8	Charlotte	Parent	YDP-L
9	Andrew	Parent	YDP-L
10	Frank	Player	YDP-L
11	William	Player	YDP-E
12	Kyle	Parent	FP-E
13	Tom	Coach	YDP-E
14	Isaac	Player	FP-E
15	George	Coach	FP-L
16	Alice	Parent	FP-L
17	Zane	Player	FP-L

### Procedure

After gaining institutional ethical approval, emails containing a project information sheet were sent out to coaches and parents, inviting them to take part in the study. Players were recruited through communication with parents, who acted as gatekeepers, and following parental consent, provided assent to take part in the study. To prevent members of the academy being aware of who else participated in the study, interested participants were asked to email the lead author directly to organize a time for an interview. Direct correspondence via email between the lead author and each interested participant allowed a connection to be developed prior to the interview (Deakin and Wakefield, 2014), and confidentiality to be maintained. Interviews were arranged with participants at a time of their convenience, with player and parent interviews being scheduled separately. Previous research has indicated that the presence of parents during children’s interviews can impact on the amount of detail children provide regarding their experiences (Gardner and Randall, 2012), thus parents were asked to leave the room when their child was being interviewed, and vice versa. Before the interview, each participant was provided with an electronic informed consent and project information sheet. Parents provided consent for their child to take part in the study, whilst children provided assent. The parent who took part in the study was the parent that deemed themselves to have the most interaction with their son’s lead coach. Participants were informed of the aims of the study and reminded their data would be treated confidentially.

#### Data Collection

Data was collected using semi-structured interviews that followed the guidelines recommended by Rubin and Rubin (2012). Due to government guidelines in place for the COVID-19 pandemic, interviews were carried out via video calling software such as Skype and Microsoft Teams. Existing literature has highlighted that the familiarity of being in one’s own environment may lead participants to feel more comfortable in talking openly (Seitz, 2016). It has also been suggested that technical issues, such as a loss of connection, may cause a loss of rapport between researcher and participant (Seitz, 2016). With this in mind, the lead author ensured that as secure a connection as possible was established with each participant before commencing the interview, and interviews began with rapport-building. The interview guide was examined by the co-authors, and then piloted with a professional soccer academy coach to ensure that the questions asked were appropriate and pertinent to the research aims.

Prior to the start of the interview, participants were asked to complete a demographic form which gathered information on the participant’s age and number of years they had been involved in academy soccer. Subsequently, the first part of the interview acted as a rapport-building period which was important for two reasons. Firstly, it was deemed necessary to establish a relationship with participants, and secondly, rapport can help relax the participants who may experience feelings of embarrassment or discomfort in being recorded (Hay-Gibson, 2009). To facilitate this, at the start of interviews with players, in order to make them feel comfortable talking, they were asked questions such as, “Which team do you support? and who is your favorite player and why?”. Questions were asked to parents and coaches relating to their history as a sporting parent or coach. Following this, the bulk of the interview addressed three main topics relative to the study aims: (1) Participants experiences of the COVID-19 pandemic. For instance, parents were asked, “What have been the main challenges for you with regards to your son’s development over the lockdown period?” (2) How have those within the athletic triangle collaborated during the pandemic. For instance, players were asked, “How have your coaches/parents been supporting you during the lockdown period?” (3) How the pandemic has impacted upon players’ holistic development. For instance, coaches were asked, “What impact do you think the COVID-19 pandemic has had on the holistic development of the players within your age groups?”. Follow up questions and probes were used to explore responses and experiences provided by participants and to facilitate the flow of the interview. Using this type of interviewing approach “assure[s] that you ask the questions to cover the overall topic and then pursue what you hear to get the depth, detail and richness you need” (Rubin and Rubin, 2012, p. 129). The final part of the interview allowed participants the opportunity to discuss any potential issues that arose during the interview or to ask any further questions they had.

The lead author conducted all the interviews and throughout the process of data collection documented personal reflections from the interviews and discussed progress of these with the co-authors. On average, parent interviews lasted 81 min (range was 60 min – 111 min), coach interviews lasted 64 min (range was 52 min – 115 min), and player interviews lasted 41 min (range was 32 min – 61 min).

#### Data Analysis

Interviews were audio-recorded, and then transcribed verbatim by the lead author with coaches and parents provided with a copy of their own transcript. Transcripts were not provided to ensure they were “true”. Rather, coach and parent participants were provided with the transcript because previous research has indicated that returning transcripts can have both affirming and cathartic outcomes among the participants interviewed (Forbat and Henderson, 2005). Moreover, by letting participants know at the start of the interview that they will be able to review their transcripts, we have learnt in previous studies that it can help to put participants at ease and increase the quality of information they share. Unfortunately, due to issues with confidentiality, transcripts were not provided to players as they would have had to be emailed to parents to be shared. Instead, players were provided with a brief overview of the main patterns found within the data.

Once interviews were transcribed, data were then transferred, sorted, and organized using NVivo software (NVivo qualitative data analysis Software; QSR International Pty Ltd. Version 12, 2019). Transcribing the interviews facilitated the close reading and interpretative skills the researcher required to analyze the data (Lapadat and Lindsay, 1999). The interviews produced 306 pages of single-spaced text, or 177,815 words from the 17 participants.

In line with an ID methodology, an approach to analysis was utilized that would capture themes and patterns within the participants’ subjective perceptions and experiences (Thorne et al., 1997). There may be similarities between individuals’ accounts, which can be identified, and the authors sought to identify and interpret these similarities. Thematic analysis (TA) was used, which provides a theoretically flexible approach to analyzing qualitative data (Braun et al., 2016). TA focuses on identifying, analyzing, and interpreting patterns of meaning within the data and follows six phases: (a) familiarization with the data; (b) generating initial codes; (c) searching for themes; (d) reviewing themes; (e) defining and naming themes; (f) writing up the report (Braun et al., 2016). In following these six-phases, the analysis was a recursive process, where the lead author continually moved back and forth, through the phases as data collection and analysis progressed.

Before coding the data, transcripts were read through to allow for familiarization with the data and notes were made with regards to anything that grabbed the lead author’s attention. Following this, using NVivo software, initial codes were identified from the data set that were of relevance to the research questions. In line with ID, it was acknowledged that the researcher’s theoretical and practical knowledge of working within an elite academy soccer club, including supporting the athletic triangle, would influence analysis (Hunt, 2009). Despite ID being a predominantly inductive and interpretative methodology, it recognizes that prior knowledge is available to the author, which in turn, can be used to guide data collection and initial analysis of the data.

Following initial coding, the process of theme development required the grouping of codes to identify ‘higher-level’ patterns that involved identifying ways of grouping codes together around a meaning or concept that they all shared. Where appropriate, codes were either removed (kept in a subfolder), conceptually expanded or reduced, or combined with other codes to create a potential descriptive theme. For example, the code, ‘recognizing the importance of sleep’ was deemed irrelevant to the research question, and did not combine with any other code, and was subsequently removed. Where appropriate, subthemes within descriptive themes were generated to identify notable, distinct patterns within a theme. For example, the two codes of ‘player ownership’ and ‘maintaining levels of independence’ were combined to make the sub-theme of ‘facilitating players’ independence’ that came under the descriptive theme of ‘Adapting practice to facilitate player and triad development during COVID-19 and beyond’.

Themes were then reviewed to ensure they formed a clear pattern, and that the proposed themes best reflected the meanings within the complete data set. At this point, data from the earlier stages of analysis were coded that may have been missed in accordance with these themes. The lead author then met with the co-authors to present the themes. The two co-authors acted as ‘critical friends’ (Smith and McGannon, 2018), allowing the lead author to engage in critical dialog and reflect upon and explore alternative explanations and interpretations in relation to the data. In line with a constructivist philosophy, that states different people may experience reality differently, engaging in conversation with the co-authors allowed the author to acknowledge that other and/or additional plausible interpretations of the data can exist (Smith and McGannon, 2018). Such discussions therefore encouraged the production of findings that reflected the data gathered, and so prompted *interpretive authority* (Thorne, 2016), enhancing the methodological rigor of the study. Once the themes had been finalized, the write-up of the study began.

## Results

During data collection and analysis, it became clear that, despite seeking to explore triadic relations, participants did not in fact spend much time working as a triad. Thus, the information provided regarding interactions across the triad was relatively limited. Rather, parents, coaches, and players instead discussed their interactions and working as dyads (i.e., parents and players, players and coaches, coaches and parents, etc.) and offered perceptions of how individual members within the triad supported one another during the lockdown. As such, in the following sections, where triadic interactions were discussed these have been shared but in the majority of situations, the focus is on dyadic interactions or individual support. Further discussion of this, along with methodological challenges associated with examining triadic interactions, are provided in the discussion.

From the analysis, three main themes relating to adaptations made by parents, coaches, and players during the COVID-19 pandemic were identified from the data: ‘Keeping everyone involved and supported’, ‘Increasing understanding of each other beyond soccer’, and ‘Adapting practice to facilitate player and triad development during COVID-19 and beyond’. These themes were considered to be recurrent across the dataset, although certain nuances were apparent in relation to the developmental age/stage of the players. Each theme contained sub-themes, which offer further insight into the experiences of interviewed participants.

### “I Just Thought I Would Call You to See How You Are”: Keeping Everyone Involved and Supported

This theme captures how those within the athletic triangle recognized the importance of maintaining communication throughout the containment measures of COVID-19. Members of the triangle, particularly parents and coaches, adapted their means of communication to ensure all other members received the support perceived as necessary during the lockdown. Specifically, three sub-themes emerged from the data: providing emotional support, the importance of interacting through various means of communication, and maintaining social connection to support wellbeing.

#### Providing Emotional Support

During lockdown, members of the athletic triangle provided support to one another to help players engage with those at the club. There was recognition from coaches and parents, that some players may become isolated and miss the chance to interact with others and engage with the remote coaching program being delivered by the club. One parent highlighted the joint effort of himself and the age group coach in reaching out to players who were drifting away from the group:

Others have sort of lent on me to be the sort of conduit between a lot of the boys in the group and pulling people together and identifying who is out on a limb or who isn’t interacting and pulling them back into the group as quickly as we can. I think me and Oliver [coach] have worked together on that as well. (Kyle, parent, FP-E)

In contrast to those in the younger age group, within older age groups, coaches seemed to encourage players to take on this responsibility themselves. For instance, as well as engaging with players himself, one coach used players whom he believed were leaders within the group to contact others to make sure none of the players from the squad were left out of any social activities or interactions:

What I did was engage with players in the group who I thought would be good social leaders…I asked one of the boys who is the admin in the group, and then I messaged the boy’s parent and asked if I could pass his number on to him to get him to add him into the group … Before I passed the group on, he was engaging a lot more with them and chatting to people and meeting up with a couple which was good. (George, coach, FP-L)

It was apparent that it was not just the players that the coaches and parents were looking out for, but also one another:

I had a call from a parent about a week ago, and he just went, ‘ah are you alright G, I just thought I would call you to see how you are?’ When I answered the phone, and he said that, I was like “ah, I wasn’t expecting that at all,” but it is quite a nice thing really … they do value what you are doing for them and their child. (George, coach, FP-L)

Similarly, coaches sought to offer support to parents whom they perceived needed it:

I’ve still tried to keep some dialog going with parents who I feel it is beneficial for…so that particular lad in question, his mum will share what she’s done … ‘I went for a run, come back and feel brilliant, I can deal with those clowns at home now’. I think that’s almost her having someone to bounce off of sometimes, to share what she’s going through at home with the lads. (Liam, coach, YDP-L)

Overall, it seemed that by providing emotional support to each other, discussions about soccer became less prevalent. As a result, interactions that occurred throughout lockdown were perceived to be more personable, supportive, and thus allowed for more open communication between parents and coaches. One coach suggested, “when they feel comfortable to share how their weeks gone with you, I think there’s a real open channel there now.” (Liam, coach, YDP-L). Throughout the interviews it was apparent that this ongoing provision of emotional support brought parents and coaches closer together and was enhanced through the use of different means of communication (detailed in the next sub-theme).

#### The Importance of Interacting Through Various Means of Communication

Participants identified various means of communication to engage with and support other members of the athletic triangle in view of government restrictions regarding face-to-face meetings. The means of communication selected was informed by the objectives for the exchange, and the perceived needs and abilities of those involved. For example, the use of video calling allowed coaches, parents, and players to continue interacting and building relationships with one another, as one player explained:

With the zoom calls … it was obviously mainly about me and what I was doing but then it also gave the opportunity to speak to my mum and dad and be like ‘how are you?’ The first part of the meeting was about me but then it was like checking on the whole family and that everything was ok … I think that’s good. It builds the relationship up in that parents can trust the coaches as well, because they play such a big part in our lives so they’re going to play a big part in their lives as well. (Daniel, player, YDP-E)

This highlights players’ awareness of the importance of the relationship between their coach and parent. This awareness seemed to be more clearly apparent among players from the older age groups.

Parents described how the use of video calls allowed them to feel invested and valued in the player development process:

They had their end of season review … they had just a small little conversation saying right you’re now going to go away, and we’ll talk about some of your strengths and weaknesses… they had a scheduled meeting the week after where we were there as well. That was quite nice that all three of us were sat round the table. (John, parent, YDP-L)

As this quote also highlights, players in older age groups were provided with more responsibility over their development than those in younger age groups, as alluded to within the last theme, and the use of technology played an important part in encouraging this.

However, despite the majority of participants discussing the benefits of video calls, there was a realization from coaches that not all parents and players were able to use video calls due to time constraints and technical issues, “We’ve got some lads who haven’t got Wi-Fi or their Wi-Fi is poor. We’re having to communicate in different ways and be really resourceful and skilful in the ways that we’re doing it.” (Liam, coach, YDP-L). Therefore, alternative means of communication were also utilized:

I’ve tried to mix my communication up. I’ve done some texts, some phone calls, some voice notes, so when I’m texting the group, I’ll do it that way as well. Some video messages as well just to try and change it up. (Oliver, coach, FP-E)

Furthermore, coaches realized that some parents were not understanding or forwarding on information to players. During the regular season, coaches would communicate information directly with players. However, the COVID-19 pandemic meant information was being communicated to players via parents. Barriers to communication included parents who worked shift patterns and were not contactable during the day, as well as issues regarding the native language of the parent. For instance, two coaches described having to translate messages before sending them to ensure key information was effectively transmitted to parents for whom English was not their first language:

There’s a Danish guy. He’s difficult in terms of having to communicate. I will write a message, and then I’ll translate it and send it to him … I called him and said, ‘are you ok me doing that [translating]?’ I didn’t want it to come across rude or condescending … He said he really liked it because, ‘some of the words I don’t understand, and you’re helping me out because you are converting it into Danish’. (Oliver, coach, FP-E)

We’ve got lads from an Islamic background. Parents speak Arabic, and we’re having to send literature out to them in Arabic now and getting translators to get it out to them because sometimes we will communicate via the lads … this will now not only help people who speak Arabic, it will help people who speak Italian, for example. That initiative now has been put into our induction pack. (Liam, coach. YDP-L)

Adapting their means of communicating, due to the COVID-19 restrictions on face-to-face contact, highlighted coaches ongoing personal development as well as adaptations to policy within the organization.

Lastly, it became apparent that how participants interacted with each other differed depending on their age. For instance, players within the YDP-L took on the responsibility of organizing interactions and sessions themselves through phone calls and social platforms such as WhatsApp, “Phone calls, texts, social media, there are so many ways in which they are communicating with each other” (Andrew, parent, YDP-L). Whereas within the FP-E, where players may not have developed the ability to initiate communication, parents were required to help facilitate interactions with other players:

I’m mates with the dad and they got given a PS4, and I said, ‘we’ve got to get the boys on FIFA and get them sorted’, so I bought the kids a 3-month membership. I just wanted Isaac to do things with his best mates, and his best mates to interact with Isaac, that part was so important for us. (Kyle, parent, FP-E).

#### Maintaining Social Connection to Support Wellbeing

Reflecting on the start of lockdown, participants across all ages discussed initial concerns with maintaining connections with players. One coach explained, ‘‘the social corner^2^ was the biggest concern…because the boys are kind of stuck away from each other, especially during the early lockdown period, and my biggest worry was that some wouldn’t engage with others.” (George, coach, FP-L). At the start of lockdown, in an attempt to support players as quickly as possible, coaches immediately looked to engage with players on a frequent basis. For some however, it became apparent that this sudden influx of support was too much:

At first there was just so many people involved…it was all good intentions, but there were just too many people…it just got too much and I just had to say in the end that’s its actually really getting to him…I think that was just the way people were trying to work it out, because it was all new. No one knew what to do. (Charlotte, parent, YDP-L)

Through trial and error, reflection, and the triad communicating such concerns, coaches began to understand how to best work with players through lockdown and provide them with the support that they and parents perceived the players required.

Participants highlighted that, at times, players struggled being away from their teammates and the academy environment, “I’ve missed the team. Like, seeing my teammates, bonding and laughing and just being with them. I’ve also missed just training with people and because they’re good players and they help me improve, I’ve missed that to be honest.” (Frank, player, YDP-L). One parent raised concerns as to whether the potential lack of communication would prove difficult for players and the development of those relationships, upon returning to the academy, “I don’t know if that has carried on. Is he still talking to those now via whatever it is, snapchat or Instagram, or have those relationships in some way stopped? It might be sort of having to restart again.” (John, parent, YDP-L). Understanding these concerns, coaches provided activities to maintain contact including group training sessions, quizzes, and cook offs. Players recognized the benefits of such contact with regards to their wellbeing:

My mates have become my stress release now, and I think that’s been a big part of coping with it. I wouldn’t say I’ve not coped well, but I think sometimes when you’re in a mood, or you just want to be by yourself, your mates can cheer you up. (Daniel, player, YDP-E)

Despite missing their friends within the academy setting, players within the younger age groups enjoyed the extra time they got to spend with their parents, time normally delimited during a regular season, “It’s been a lot nicer to spend time with my family… normally we don’t get to spend that much time with each other…but it has made our relationship better.” (Isaac, player, FP-E).

Parents also referred to the benefits of the activities set out by coaches in attempting to maintain the social connection amongst players, “I think those zoom sessions with the yoga, and the ball mastery…he looked forward to doing them as he could see his friends and the coaches were there… so he liked it.” (Alice, parent, FP-L). Players within the older age groups also took ownership of connecting with their friends not just within soccer, but also with friends outside of the academy environment. Much of this engagement occurred on games consoles, which provided a space for all players to spend time with friends, independent from their parents and coaches, “I think having an Xbox and facetiming my mates and speaking to them helps that I can still communicate with people.” (Daniel, player, YDP-E). Parents, who noted that they would typically try and limit their child’s use of gaming, discussed the benefits that this provided during the lockdown period, “He has that interaction, and we don’t stop that interaction because in this period of COVID, that’s good, because you can hear him laughing and talking to them and playing … so we’ve not tried to stop that.” (Andrew, parent, YDP-L).

The majority of participants highlighted that the space to connect provided by coaches and players would prove valuable in maintaining the cohesiveness of the team when players returned to training:

It has helped. Especially when we come back, it’s not like we haven’t seen each other for six- months, we’ve already seen each other through it, so it won’t be that much of a shock when we get back really… If we hadn’t seen each other in a long time, everyone might be a bit disjointed really. (Zane, player, FP-L)

In addition, one parent highlighted that through the athletic triangle regularly connecting, their child was able to adapt to the difficulties faced at the start of the pandemic. This led to effective future working amongst the triad, as well as increased levels of player wellbeing:

I remember the coaches saying, ‘look is he alright?’… they’d noticed he wasn’t very happy. I said, ‘that is exactly how he is at the moment, and we’re really struggling with him. He’s moody, he’s hard to motivate, and he doesn’t really want to do anything’. They realized that as well, so we had that discussion. A week or two after that he started to get better and within a couple of weeks me and the coaches were just saying that he was a different boy. He’s motivated, he’s more positive, his mood had lifted, and since then he is just doing everything that he needs to be doing and enjoying it again. (Charlotte, parent, YDP-L)

To conclude, this theme illustrates how members of the athletic triangle used various means of communication to connect with and support each other during the lockdown period.

### “Now They See Me as a Normal Person”: Increasing Understanding of Each Other Beyond Soccer

This theme captures how interaction with others in the athletic triangle during lockdown helped participants understand one another beyond just soccer. Specifically, two sub-themes emerged from the data: getting to know the person and awareness of roles and responsibilities.

#### Getting to Know the Person

Participants suggested that through interactions between the different members of the athletic triangle that they were able to gain a deeper understanding of the person in front of them, including aspects of their personality, home life, and interests outside of soccer:

It showed us a lot of character traits of some of the boys. Some of the players have surprised us … we’ve got to know the boys socially… you know the name of the dog, you know they’ve got two sisters, and you’re asking them how they are and how’s this going … you’ve found out a lot about the kids. (Tom, coach, YDP-E)

A consideration reinforced by one parent, who through her interactions with their son’s coach, stated:

They’ve learnt so much more about the boys individually because of lockdown, rather than just seeing them in their academy kit playing football. They feel they know them as different people now, and to me, that can only be a good thing. (Alice, parent, FP-L)

In attempting to understand why this pattern emerged amongst coaches, it was highlighted that time freed up during lockdown was more challenging to find within the pre-lockdown academy environment, “You don’t usually have lots of time for those little conversations, but they’re the most important bits, and we’ve had loads of time over the last few months.” (Tom, coach, YDP-E).

As a result of this increased understanding of individuals, participants believed such interactions during lockdown have served to enhance the relationship between the coach and player, as one player stated:

Where they’re calling us to see how we are, what we’ve done, how schoolwork is getting on … I think it’s helped with your coaches; you’ve become closer. You understand each other better, when you communicate with them, you can speak to them more. (Daniel, player, YDP-E)

This was a view supported by one coach, who when asked about his relationship with players during the lockdown, remarked, “I’ve really made an effort to really get to know what he likes, what he doesn’t like, what he’s doing. Hopefully when we go back, because he’s quite standoffish and withdrawn, I’m hoping we’ve got a bit more connection.” (David, coach, YDP-L). One coach provided an example of how, through further questioning, he was able to gain a greater understanding of the home life of one of his players, “I thought, ‘I’ve got to do a little bit of digging deeper here’ … I found out that the parents worked nights, and they had been struggling to get in contact during the day because they’d been sleeping” (George, coach, FP-L). This in turn informed how coaches may best work with this individual moving forward:

‘This boy is going to be hard to get hold of when he comes into your age group, but this is what I found worked and put those in place, and these are the reasons why he is going to be hard to get hold of’. I would have hated him to go into that age group, and then this new coach can’t get hold of him, and then already they’re off on a bad foot without understanding why. (George, coach, FP-L)

Interestingly, one player went as far as to state that were it not for the pandemic, he did not think his relationship with his new coach would have flourished to the extent that it has:

If we were still at [names club] we wouldn’t have done that, because we would be at training … but I think we’ve done well over zoom to get to be able to know each other more before we go back into training…if you know them well, it will boost your confidence.” (Zane, player, FP-L).

As a result of the interactions parents had to facilitate during the lockdown, coaches believed parents had also developed a greater understanding of them as people, not just solely someone who coaches their son:

It has been a lot more individualized and personalized. They’ve sort of seen us as people and not just their coach…we have families, we have the same stresses that you’re having…it has changed a bit over the last few months…which I’m sure will have its benefits this year. (Tom, coach, YDP-E)

This increased understanding was particularly notable among parents of younger age group children, where parents continued to play a more active role in their child’s development. One coach believed that this understanding would lead to parents potentially changing how they behave and interact with their coach, “By them understanding that you are actually a human being, and that you have got feelings and emotions as well, that they will think about what they’re doing before they do it.” (George, Coach, FP-L). This development becomes increasingly important when considered alongside a quote from a parent with regards to the relationship some parents seem to have had with their coaches during the regular season:

They’re scared because they think they’re going to cut their son from the academy. Even if they’ve got a good or a genuine concern… Oh, I wouldn’t say scared, but they don’t know how to approach them… There are still parents in some age groups who haven’t got the coach’s number. They haven’t got a clue. They don’t know. They just drop them off, pick them up, and they’re gone. (Andrew, parent, YDP-L)

Lastly, responses indicated that this period has raised coach’s awareness of the importance of engaging with and understanding parents. In discussing the workings of the triad, one coach stated, “It’s not just working with the individual player, it is understanding the parent values and the messages that are being reinforced at home. Having some level of understanding around them is so important to me.” (Liam, coach, YDP-L). Supporting this, it was mentioned that the past few months have allowed coaches to develop that understanding, “you’ve got to know their parents and the importance of that, connecting with them on a sociable or a more personable level. I think there has been lots of learning to do around that.” (Tom, coach, YDP-E). Players recognized how the enhanced interaction and understanding between their coach and parent had led to benefits within the coach-parent relationship, “For them [parents] it’s also becoming confident in being able to talk to your coaches as well. I think that’s important and I think that’s been built over lockdown.” (Zane, player, FP-L).

Overall, it became evident that the period in lockdown has provided parents and coaches with greater knowledge of one another, “it’s probably the biggest thing for me that I’ve taken from the lockdown is gaining and forming better relationships with the first layer of the players family.” (Oliver, coach, FP-E).

#### Awareness of Roles and Responsibilities

It became apparent during the pandemic that those within the athletic triangle were carrying out more roles than would usually be the case. Some coaches felt they were taking up the role of not just a coach, but also a friend and/or an educator. In contrast, parents started playing the role of a coach by facilitating soccer sessions and providing feedback to their son. Through this, participants felt they have become a lot more aware and understanding of the role that one another plays. One coach remarked:

Lots of parents have said to me ‘I actually now understand how hard you work and how difficult it is to coach them throughout the weeks and months and giving them the right detail and understanding them.’ It’s helped with their understanding of how much work goes in for their boys. (Oliver, coach, FP-E)

Parents, particularly among younger age groups where they are more involved in supporting their child’s sporting development, described how they sought to facilitate training with their son. Specifically, it was fathers who facilitated the technical, tactical, and physical aspects of training, “Watching the clip, going out and practice, and then when I’ve finished work, we can go and refine some of the things that from a technical perspective that he needed to work on.” (Kyle, parent, FP-E). Players also commented on how parents, particularly fathers, had been helping them with their soccer objectives:

He told me to watch De Bruyne and his crossing technique, and then we would go to the park or something, and we would do it, and then he would be like, ‘what did you do well? and what do you need to work on?’. (Zane, player, FP-L)

Undertaking such roles has meant that fathers have become more informed and understanding of their son’s sporting development. In facilitating this, coaches played the role of an educator to both player and parent when communicating aspects of the training program during the lockdown, “We have to educate them as to why, even to kingdom come they might not still value it, but I think our jobs now is to help and support and educate, rather than be the enforcer” (Liam, coach, YDP-L). This quote highlights how the potential role of a coach has changed over time, and that the current pandemic has highlighted this. This increased understanding allowed parents to provide the necessary informational support, alongside the emotional support alluded to in the first theme, that was needed during the lockdown period:

When we’re explaining to the boys their new programs, they’re on the calls, and they’re supporting…then they’re also able to nudge their lad a bit because they are aware of the program. They have been quite supportive in getting the boys to do the stuff. (Tom, coach, YDP-E)

Similarly, one parent highlighted that having such knowledge about technical and physical training programs helped him to learn more about aspects of his son’s development. He believed this allowed all members of the athletic triangle to feel a part of the development process:

It’s been a huge advantage for me in this process. Being able to hear it and understand it, because Oliver won’t just talk about the successes, he will talk about the things you need to work on … the feedback triangle was complete. (Kyle, parent, FP-E)

Additionally, players believed that the increased involvement that parents have shown over the lockdown period would facilitate their parents understanding of the way they played the game, leading to more agreement across the athletic triangle. When asked if he believed that lockdown might have given his parent more of an idea about how he and his team play, one participant responded:

I think he’s [my dad] started to understand my playing style and that will help … if his opinion on something was different to mine, he might understand maybe how I would think of playing that ball would be different to his because of how my playing style is different or how [names club] have taught us differently. (Daniel, player, YDP-E)

Lastly, in line with the sub theme ‘facilitating player independence’, it became clear that players were undertaking the role of a coach within their own development during the lockdown as one coach stated, “they’ve understood and took ownership of what their needs are to develop and get better and they’ve gone about their own training.” (Oliver, coach, FP-E). One player described how, on the back of increased analysis sessions, he wanted to continue understanding and developing his skillsets, “they’re the main ones I want to carry on doing, looking at previous games…analyzing what I did well and what I didn’t and just practizing it outside of [names club] as well.” (Zane, player, FP-L).

In summary, this theme highlights how increases in engagement amongst those within the athletic triangle, as highlighted in the first theme, have helped participants develop a greater understanding of others within the triad. Also, it emphasizes how interactions have led to an enhanced awareness of the roles one another plays and have subsequently adopted, which participants think will aid in the holistic development of the player as they progress through the academy.

### “We Need to Move With the Times”: Adapting Practice to Facilitate Player and Triad Development During COVID-19 and Beyond

The last theme captures how lockdown led parents and coaches to provide an increased sense of independence within players. Furthermore, participants discussed how interactions during COVID-19 enhanced the interaction of the triad and the importance of learning from this when moving out of lockdown. Specifically, two sub-themes emerged from the data: facilitating players’ independence and enhancing the triad moving forward.

#### Facilitating Players’ Independence

Participants discussed how players were provided with opportunities to take ownership over their development and how they would look to continue to enhance independence within players post-lockdown.

Parents and coaches discussed how they looked to encourage independence within players, through providing them with greater ownership over their development through lockdown, “When we have done zoom sessions online, and ball mastery sessions, I would give a lot of ownership to them, getting them to show what they can come up with rather than me leading it.” (George, coach, FP-L). Another coach shared a similar example, “Tomorrow is a player-led session. So, the players have got to come to the session with a 1-min activity, and they will become the coach for 1 min.” (Oliver, coach, FP-E). Within the YDP, parents discussed how they were already allowing their son to take control over of their development, “I have less interaction now with Frank … we have to make him stand on his own two feet and find out himself what it is all about without me having to remind him all of the time” (Andrew, parent, YDP-L). Similarly, in talking about their son’s relationship with his coach, Rachel stated, “I feel like it’s his space … he is coping with it all. He tells me if he wants me to help him or dib in if you like … He’s old enough to do it all himself.” (Rachel, parent, YDP-L).

There were similar responses from parents who were committed to allowing their children to take ownership of particular elements at home:

I’m full-time [working], so he has to do his dinner himself, which he has never done before. He has learnt how to do beans on toast and things…he has learnt how to help himself independently … he’s just developed a lot of independence skills, and self-management, and he’s a lot happier. (Charlotte, parent, YDP-L)

Reflecting on his practice, one coach believed the opportunity for players to develop independence was not provided enough during the regular season:

We are a little bit guilty of that, especially in this environment, where we do absolutely everything for them. We schedule everything. We transport them, we email them, we call them, we text them, we put plans in place, we take them on tour … we do absolutely everything for them. (Tom, coach, YDP-E)

Another coach discussed how he will look to use some of ways he has worked during lockdown upon returning to the academy:

It’s the way that we speak to them in terms of coaching engagement and coaching behavior. Not being so commanding with them…Do you know what, these boys have a lot of knowledge, and using things like question and answer and guided discovery and allowing them to solve problems a little bit more will help develop independence in terms of thinking. (George, coach, FP-L)

Comments from players supported the notion that they had become more independent with regards to their soccer development during the lockdown. As a result, this led to them making their own decisions, and taking more responsibility for their soccer development:

I woke up three times in a row at 6 o’clock to go up to the field when it wasn’t boiling hot. They gave us responsibility to be like, ‘you can manage it how you want. If you want to go out in the boiling heat, it’s up to you, but if you want to make the effort to get up when it’s still cool, you can do’. Instead of being like you’ve got to do your run between 11 and 1, it gave you the responsibility to do whatever you wanted. (Daniel, player, YDP-E)

This increased independence during lockdown enhanced players’ levels of motivation with one player stating that this would impact their approach to their development post-lockdown, “I’ve got time to focus, and work on my own improvements…it’s given me the motivation…when I go back, I want to be flying…it’s given me more time and more thinking to make myself as good as I can be.” (Frank, player, YDP-L).

Furthermore, through placing more ownership on players, as well as placing them into a variety of situations, participants described increases in players levels of confidence, as one coach described:

There was a boy in the last age group, and he would come on to the first call, and he wouldn’t say a lot with a lot of people in there. You would speak to him and get like a couple of word answers. Then, it was in an analysis task, when he was presenting back some clips, and he was on fire. You couldn’t get him to stop talking, and you could tell that he felt a lot more comfortable and confident doing it. (George, coach, FP-L)

This increase in confidence was also supported by one parent, who remarked, “His confidence has grown from that, confidence around talking on facetime…talking on zoom calls with no football. All those confidence levels have gone up.” (Kyle, parent, FP-E). Subsequently, it was hoped that this developed independence and newfound confidence would continue as players returned to training following the lockdown, “I hope he’s able to box it and take it back into the training ground, but I suppose that’s down to how the coach takes all of these learnings and brings them back into the training ground” (Kyle, parent, FP-E). A thought reinforced by one coach, who wanted to make sure this focus on player independence continued to be applied post-lockdown, “it’s making sure that we’re still autonomy supportive, and we’re still allowing them to be independent when they come back in.” (George, coach, FP-L).

#### Enhancing the Triad Moving Forward

Participants made reference to how the period in lockdown has given them time to reflect on how they might apply what has been learnt during COVID-19 to support workings within the triad post-lockdown. For instance, coaches discussed how utilizing new means of communication like video calls, as alluded to in the first theme, could act as a way of increasing parents’ understanding of their son’s development process. Participants referred to particular issues faced within the regular season when it came to reviews and parents evenings, “I remember driving there and you get a text from one of the parents saying, ‘don’t worry about putting your foot down to get here, they’re running 45 min late’ (John, parent, YDP-E). Making reference to such issues, coaches highlighted the practical benefits that using such means of communication would bring in the future:

I’ve been able to share screen with parents and the players and do their reports from home. I don’t have to drag them to [names club] to have a 15-min meeting with them. So, I think, when its report night, instead of dragging them in, we can give them the option, ‘do you want your report night over Zoom, or do you want your report night face-to-face?’ (Tom, coach, YDP-E)

This was a view supported by parents, who when asked what practices they would like to see continue, stated, “Definitely these kinds of communications. You know via Zoom, Teams, Skype whatever it is. I think those things would be really good to continue with and having reviews via that” (John, parent, YDP-L). Parents believe utilizing such means of communication would lead to stronger relationships between the coach and player and that such interactions have allowed players to feel more comfortable giving feedback to coaches than they might have done pre-lockdown, “I think that would be a really good thing thinking about that personal relationship with them … and on a zoom meeting I think they [players] probably give more information out.” (John, parent, YDP-L).

Coaches suggested that moving forward, information gathered about individuals within their age group would prove valuable in supporting varied aspects of their soccer and psychosocial development, “I now know why some boys are the way they are and what I might need to do to get them better and improve them in the psych corner and the social corner.” (Oliver, coach, FP-E). Coaches stated that this would help shape work with players following their return to the academy, “we’ve found out a lot about the boys, about their drive, their self-motivation, their work ethic. So that has helped shape their individual plans.” (Tom, coach, YDP-E). Returning to the regular season, coaches discussed how they might look to integrate such interactions more intentionally into their practice:

There is a lot of time when, say a boy gets in early, you’d ask them to use their time wisely and go and practice. Whereas actually, using your time wisely might just be having a chat with them. It might just be generally finding out a little bit more about them, ‘what have you been up to today?’, and then just having a bit more of a laugh, which we do anyway, but having a bit more of a conscious effort to it. (George, coach, FP-L)

When training schedules do not allow coaches enough time to have individual conversations with players to gather such information, again the use of video calls could prove useful:

Because of the back-to-back nature of our job, doing back-to-back sessions, the boys come in and they’re working all the way through, you hardly get to speak to people. So, organizing a time where we can go and chat to players over zoom, like we have now over an individual level, to see how they’re getting on. (George, coach, FP-L).

This view was supported by parents, who believed that this form of communication had already benefitted the triad and could be used to help coaches further develop relationships with players and parents when the time does not permit within the academy environment:

Maybe use these zoom calls as a catch up with parents and the children, to see how things are … because obviously, it is difficult to speak to a coach on training night during football. It might be something to think about, to have that interaction with the parent and the child outside of football just to sort of catch up with them. (Alice, parent, FP-L)

One parent highlighted how using such means of communication could be of benefit during other adverse events to help coaches maintain connection with players, “it can also be a perfect way of coaches keeping touch with players when they’re injured. To see how they’re doing and to Skype them” (Andrew, parent, YDP-L).

As this theme highlights, it is important that coaches, and other stakeholders within the organization use what has been learnt during the lockdown to help facilitate the future workings of the athletic triangle. It was clear from participants responses that there was a willingness to make use of the positive aspects to come out of the pandemic, “I think it will be taking a sideward step if we went back to normal and we just ditched it all. We need to move with the times.” (Oliver, coach, FP-E).

## Discussion

The overall purpose of this study was to explore the perceptions of players, parents, and coaches regarding how they adapted their interaction and collaborations during the COVID-19 lockdown and how such adaptations can be used as English soccer academies, and other youth sports clubs and organizations, return from lockdown. It should be noted that although the authors aim was to examine triadic interactions, what became evident from the data was that there was actually limited triadic engagement. For instance, few stories or experiences appeared to be shared between, and subsequently discussed by, all members of the athletic triad. The absence of triadic interactions and collaborations was, in itself, an interesting finding and points to the potential challenges of trying to facilitate interactions between three parties when such interactions have to happen remotely. Nevertheless, the data provided substantial insights regarding the functioning of dyadic relationships within the broader triad, as well as perceptions of what was deemed to be helpful by different triad members during the pandemic.

Findings from the current research highlight the positive adaptations observed by those within the athletic triangle over the lockdown period. Interaction between particular dyadic relationships within the athletic triangle increased, which as a result, led to the development of stronger relationships. Furthermore, such interaction allowed for an increased understanding of other members of the athletic triangle and their roles within the players’ holistic development. Lastly, participants described the importance of utilizing such adaptations as the club moved out of lockdown. Through adapting and continuing to interact, all members of the athletic triangle were able to support the wellbeing of each other and facilitate their personal and professional development.

In line with the notion that dyadic relationships begin, grow, and are maintained within a larger social network (Sprecher et al., 2002), the current findings support previous research by Jowett and Timson-Katchis (2005) who suggested that more involvement from parents may be beneficial for player-coach relationships. Increased interaction allowed parents to provide coaches with key information about their child’s mood, wellbeing, and general progress, as well as feedback from the player on sessions that were being delivered. Not only did this information allow coaches to adapt their way of working for certain individuals, it also provided the opportunity for the coach-athlete relationship to grow.

The importance of support provided by those during the lockdown, and the connections developed with each other, can be understood through the model of thriving through relationships (Feeney and Collins, 2015). It is well recognized that close relationships are linked to health and wellbeing across an individual’s life (Pietromonaco and Collins, 2017). During adverse events, such as COVID-19, close relationships can protect individuals from the negative effects of stress (Uchino, 2009). In their model, Feeney and Collins (2015) argue that people will emerge from adverse life events stronger than they were before the event through the support of others who help people strengthen and assist them in rebuilding. They suggest that social connection, a critical factor within the current research, helps individuals maintain a positive effective balance, facilitate emotional regulation, and build resilience. Furthermore, the researchers argue that close relationships can alter the way in which individuals appraise a situation or event, and ultimately cope with perceived stressors (Feeney and Collins, 2015). Findings highlighted how the support received from others in the athletic triangle had a positive impact on how individuals managed the lockdown period. The implications of these findings become particularly pertinent when considered alongside the many potential adverse events those in the athletic triangle may experience during their academy journey (i.e., release, deselection, and team performance; Reeves et al., 2009; Harwood et al., 2010; Dixon and Turner, 2018). The current research gives support for the importance of close relationships and the support provided during the COVID-19 pandemic. The findings highlight the benefits that social connection can provide an individual as they attempt to navigate their way through potentially stressful events. Future research should examine the influence of triadic relationships on the performance and wellbeing outcomes of players following adverse events in academy environments.

The findings also offer further support for the importance of shared and communicated goals between parent and child (Knight and Holt, 2014). The increased interaction between the parent and coach dyad allowed coaches to regularly provide information on aspects of the training program to parents. Parents highlighted that increased communication allowed for an enhanced understanding of their child’s current soccer experience and goals, a key factor underpinning optimal parental involvement within sport (Knight and Holt, 2014). Previous research has highlighted that parents can feel excluded by coaches, meaning they feel they are unable to facilitate their sons’ development (Harwood et al., 2010). Existing literature has also suggested that parents lack of knowledge of the sporting system they are involved in limits the support they can provide their children and speak of a need for more guidance and support from organizations and coaches (Harwood and Knight, 2009; Knight and Holt, 2013). Within the current research, parents described an increased understanding of their child’s sporting development, such as understanding the reasoning behind certain sessions. Parents stated the information provided from coaches allowed them to feel a part of the communication loop, enhancing their understanding of what their sons’ current goals were and how they might help support these.

Existing literature has highlighted the uncertainty parents have in approaching coaches as another potential stressor (Clarke and Harwood, 2014). Parents within the current research made reference to such uncertainty occurring during the regular season. However, it was emphasized that the increased interaction between the coach and parent during the lockdown had allowed parents to feel more comfortable making contact with their sons’ coach and that this would continue to be the case as they returned to regular training. From a coaches’ perspective, coach stressors identified within youth tennis highlighted the lack of respect parents have for the role of the coach as a substantial stressor (Knight and Harwood, 2009). Current findings suggest that increased interactions between coach and parent allowed parents to better appreciate their coaching role, and also their identity as a human being who experiences similar emotions to themselves (Knight and Harwood, 2009). Coaches anticipated that parents have become more aware of the impact their behavior and interactions have on a coach’s emotional state and will demonstrate greater respect. The current pandemic has forced coaches and parents to actively engage with each other, leading coaches and parents to become more aware of how they treat one another. Through enhancing awareness of those people around the player, such as their parents, coaches will become more sensitive to the ecology of the player (Henriksen et al., 2010), which should facilitate players’ holistic development.

The current findings highlighted how coaches and parents facilitated players’ desires to remain socially connected. Existing literature has revealed that those in late childhood require interaction with other young children to develop their socioemotional wellbeing (Thomson and McLanahan, 2012; Hastings et al., 2015). Furthermore, adolescents are at a period in their lives where there is an increased need for peer interaction (Lam et al., 2014), which is important for crucial functions in mental health (Orben et al., 2020). Despite concerns raised by parents and coaches, players used digital technology (i.e., video games, Facetime, WhatsApp) as a means of maintaining connection. The use of such technology has been linked to increases in wellbeing as well as helping to maintain personal relationships (Burke et al., 2010; Ellison et al., 2014). In their review of the impact of COVID-19 on adolescents’ mental health, Orben et al. (2020) argued that the use of digital technologies may mitigate some of the negative effects of physical distancing. The findings from the current study provide evidence for the benefits of digital technology in maintaining social connection among adolescents. Therefore, it is suggested that, in line with safeguarding measures within academies, the use of such technology to maintain connection may prove beneficial when players are experiencing a lack of physical interaction within certain relationships (e.g., long-term injury). Findings highlighted that means of communication was adapted for, and differed between, age groups. For example, in line with psychosocial development, interactions between younger players (e.g., FP-E) occurred through parents, whereas older players (e.g., YDP-L) organized their own interactions with their peers and coaches. It could be argued that through their increased desire for, and development of independence (Steinberg and Silverberg, 1986), as well as their increased confidence and enhanced communication skills, that adolescents were able to organize such interactions themselves. However, connection to parents continues to be of great importance for those in late childhood, as they continue to learn how to initiate and maintain friendships (Rice, 1998). It became apparent that those within the FP required further support from their parents in initiating such interactions. Furthermore, in line with previous findings, parents within the younger age groups took on a more active role in their child’s soccer development during lockdown (Fraser-Thomas et al., 2008) compared to parents in the older age groups whose involvement was limited (Knight et al., 2010).

Furthermore, the findings identified that players developed an increased motivation toward their development as a player during the lockdown. According to Deci and Ryan’s Self Determination Theory (SDT; Deci and Ryan, 2000), the quality of an individual’s motivation, in this case the player, is influenced by the satisfaction of three basic psychological needs (BPN): autonomy (the need to experience choice), competence (the need to feel competent and have capacities to accomplish goals), and relatedness (the need to experience interpersonal connection and caring; Deci and Ryan, 2000). The findings highlight that players BPNs were fulfilled during this period in lockdown. For example, players discussed having control over their development (autonomy), were able to meet challenges set by coaches (competence), and that they felt valued and connected to others (relatedness). Satisfaction of such needs has been shown to positively predict self-determined motivation, leading to enhanced wellbeing (Jowett et al., 2017). Therefore, coaches and parents alike should seek opportunities to support the players’ experience of the BPNs to nurture the most high-quality forms of motivation and engagement in their development, to enhance both wellbeing and performance. For instance, coaches could look to provide increased independence within development plans or provide choice to players over particular aspects of training by allowing players to have their say on particular training drills.

Lastly, previous research has highlighted that fathers are perceived as being more involved and more influential in their children’s sports participation than mothers (Babkes and Weiss, 1999; Lavoi and Stellino, 2008). In the current research, participants described that fathers took on the role of facilitating technical, tactical, and physical aspects of training. However, it must be noted that half of the parents in the current study, who labeled themselves as the most involved parent, were mothers. This could be due to the increased female role models and popularity of the women’s game (Wrack, 2019). Therefore, organizations should be encouraged to further involve female parents when discussing technical, tactical, and physical aspects of a player’s sporting development.

### Limitations

There are certain limitations to the study that should be considered when applying findings or looking to extend them. Firstly, there have been varied responses of soccer clubs in the face of the COVID-19 pandemic. For example, as of April 20th, many clubs placed staff on ‘furlough^3^’ following announcement of the British government coronavirus job retention scheme, while others continued to pay employees their full salary. The club from which the participants were obtained did not place employees on furlough, and unlike those who did, were able to continue to communicate with players and their parents throughout the lockdown. Therefore, the experiences of those interviewed would differ greatly from those at clubs who experienced furlough. Secondly, there were difficulties in interviewing players who were of a younger age which, at times, led to less insightful responses. The open-ended nature of the questions may have proved confusing or overwhelming for the younger participants who are in the midst of developing cognitive skills such as considering hypothetical situations, and realizing others have views that are different to their own (Mack et al., 2009). As highlighted, triadic interactions and experiences were limited within the data collected. On reflection, it was apparent that trying to capture triadic interactions proved challenging. A singular interview with each participant meant it was not possible to gain further insight from a member of the triad once they had already had their interview. Carrying out multiple interviews would have allowed the researcher opportunities to build on information given in previous interviews and to further explore interactions and experiences with each member of the triad to help better capture triadic workings over a period of time (Byrne, 2001). Furthermore, utilizing alternative methodologies such as daily diaries (e.g., written journal entries or audio voice notes) would have allowed for participants to reflect upon interactions with other members of the athletic triangle as they occurred and provided data in real time (Swainston et al., 2020). Despite seeking variation in the sample of participants, those that volunteered to take part in the study may share common characteristics different to those parents who chose not to take part or who did not respond to the invitation sent out by the lead author. Therefore, further research utilizing participants from different populations of parents, coaches, and youth soccer players, as well as those from other sports, may provide further knowledge of the discussed experience. Lastly, it became apparent that there were nuances between the younger and older age groups. Understanding this, future research could look to investigate the differences between age groups as they experience similar transitions.

### Applied Implications

Research examining parent-coach interactions in sport is limited (Knight and Gould, 2017). This study extends the current literature examining those relationships within athletic triangle, by providing an understanding of how parent and coaches can positively work together to facilitate players’ holistic development. Implications can be drawn from this study that are of central importance to parents, coaches, and organizations when looking at how to enhance the workings of the athletic triangle to facilitate enhanced wellbeing and performance. Such implications are suggested:

1. Continued communication: Findings highlighted that the more parents and coaches interact, and the more information that is subsequently shared, the more consistent messages will be across the athletic triangle. This in turn will provide clarity of message for the player with regards to their soccer and psychosocial development. Therefore, opportunities for parents and coaches to regularly engage with one another should be encouraged and various means should be used to facilitate such communication.
2. Getting to know the individual: Players, coaches, and parents all discussed the benefits of getting to know one another as individuals beyond their sporting role. Coaches should use opportunities before and after training to get to know those they are working with, in turn developing these relationships, and understanding how to best work with each individual. Inductions at the start of every season introducing parents to key members of staff, including coaches, will allow parents to start developing their relationship with their son’s coach. The development of relationships will provide the support required to thrive during adversity.
3. Reflect and utilize the key skills learnt: The pandemic has led to the development of skills across all participants. Understanding how, and why, to use new tools, like Zoom, can be important for coaches in allowing regular communication to continue (e.g., player reviews). Players should be encouraged to reflect back on key skills learnt (e.g., confidence in speaking in front of their peers, increased independence, leadership skills) and how these can be used both inside and outside of soccer. Similarly, reflecting with parents and coaches on how these were developed during lockdown, and then providing joint educational support in how to develop such psychosocial skills, would be worth considering.

## Conclusion

Overall, the present study highlights the way in which those within the athletic triangle collaborated with one another over the COVID-19 pandemic. The study focused on the adaptations made throughout the COVID-19 lockdown and the importance of continuing the enhanced means of working as academy soccer returns from the pandemic. The findings highlight the importance of parents and coaches regularly interacting in order to provide relevant information regarding players’ holistic development and to build relationships. Further research could look to understand how parents and coaches apply what has been learnt during the lockdown moving forward, to positively influence a player’s development through their academy journey.

## Data Availability Statement

The raw data supporting the conclusions of this article will be made available by the authors, without undue reservation.

## Ethics Statement

The studies involving human participants were reviewed and approved by Wolverhampton University Faculty of Education Health and Wellbeing Ethics Panel. Written informed consent to participate in this study was provided by the participants’ legal guardian/next of kin.

## Author Contributions

JM was responsible for collecting the data, leading data analysis, and the process of writing the manuscript. TD and CK were responsible for supporting the development of the research question, the data analysis process, and the writing of the manuscript. All authors contributed to the article and approved the submitted version.

## Conflict of Interest

The authors declare that the research was conducted in the absence of any commercial or financial relationships that could be construed as a potential conflict of interest.

## Footnotes

1 Each academy across the English Premier League and the English Football League is ranked between 1 and 4 (one being the highest and four being the lowest) based on criteria including productivity rate, training facilities, coaching, education and welfare provisions.

2 The Football Association (FA) 4 Corner Model suggests that player development needs to be looked at from a technical, physical, psychological and social point of view.

3 For further information on furlough arrangements please see https://www.gov.uk/guidance/claim-for-wage-costs-through-the-coronavirus-job-retention-scheme.
